# Visual inspection with acetic-acid (VIA) service utilization and associated factors among women in Hawassa city, southern Ethiopia: a community based cross-sectional study

**DOI:** 10.1186/s40695-021-00065-4

**Published:** 2021-07-23

**Authors:** Getinet Kassahun Azene

**Affiliations:** Department of Midwifery, College of Medicine and Health Science Hawassa University, Hawassa, Ethiopia

**Keywords:** VIA screening, Women, Cervical cancer, Urban community

## Abstract

**Background:**

Reducing cervical cancer mortality and morbidity using visual inspection with acetic acid (VIA) is a primary option, particularly in resource constrained countries. Although VIA screening is a priority program in Ethiopia, there is limited scientific evidence on prevalence of VIA screening service utilization and factors influencing screening practices in the community. This study aimed to assess the magnitude of visual inspection with acetic-acid (VIA) service utilization and associated factors in an urban community among women in Hawassa city, Southern Ethiopia.

**Methods:**

This community-based cross-sectional study was conducted among women aged 30–49 years old who were residents of Hawassa city. The study population (*n* = 419) was recruited using a multistage random sampling technique. A pretested and structured interviewer-administered questionnaire was used to obtain information on sociodemographic characteristics, reproductive and behavioral variables, awareness of cervical cancer and VIA screening, and VIA screening practices. Multivariate logistic regression models were used to determine factors associated with VIA screening service utilization.

**Results:**

A total of 411 women aged 30–49 were interviewed with a response rate of 98.1%. The visual inspection with acetic-acid (VIA) screening service was utilized by 85 women (20.7%). Multivariable logistic regression analysis showed that use of VIA screening service was significantly associated with older age (adjusted odds ratio (AOR) = 4.64, 95%CI: 2.15–10.01), having a history of sexually transmitted infection (STI), (AOR = 3.90, 95%CI: 2.02–7.53), having awareness about cervical cancer and VIA screening (AOR = 3.67, 95%CI:1.68–8.04), self-perceived susceptibility (AOR = 3.52,95%CI:1.74–7.13),receiving information from health workers (AOR = 4.519, 95%CI: 1.686–12.114) and having received community health education from health extension workers (AOR = 6.251, 95%CI:2.994–13.050).

**Conclusion:**

Self-reported use of VIA screening was low in the study area. Age of participants, history of STI, awareness of cervical cancer and VIA screening, self- perceived susceptibility, receiving information from health workers and community health education from health extension workers were associated with increased prevalence of VIA screening service utilization. These findings suggest that educational and clinical interventions at the community levels and in healthcare facilities should be strengthened to improve cervical cancer risk knowledge, and to encourage women to seek cervical cancer screening in approved settings to order to increase utilization of the service.

## Introduction

Cervical cancer has the third most frequent incidence rate of all cancers in women globally, with an estimated 569,847 new cases and 311,365 deaths per year [1]. In 2018, the East African region had the highest incidence of cervical cancer, with approximately 52,633 new cases and 37,017 deaths [2]. It is the second leading cancer after breast cancer amongst Ethiopian women, with an estimated 6294 new cases and 4884 deaths per year [3]. Despite high cervical cancer morbidity and mortality in East Africa, the above figures are under estimated due to limited or no access to screening services and lack of a population-based cancer registry [4].

Cervical cancer is caused by persistent or chronic infection of the human papillomavirus (HPV); a sexually transmitted infection [5]. The most prevalent oncogenic types among women in the East Africa are HPV 16, followed by HPV 18 and HPV 52 [5]. Even taking into account the challenges of obtaining vaccines against these three HPV types in the region, vaccination against HPV 16 and 18 will reduce the future burden of cervical cancer in sub-Saharan Africa [6]. Other risk factors for the development of cervical cancer include: first time sexual intercourse at an early age, multiple sexual partners, high parity, smoking, long term oral contraceptive use and HIV infection [7].

When detected at an early premalignant stage, cervical cancer is largely preventable [8]. In high-income countries, cytology-based screening (the Pap test) remains the most popular screening technique for cervical cancer prevention. But, this method is limited in low-resource countries, including Ethiopia due to delayed results, poor follow-up, lack of laboratory infrastructure and too few trained cytotechnicians or pathologists [9, 10]. To be effective HPV testing requires a well-resourced, advanced healthcare infrastructure and appropriate patient follow-up [11].

In contrast, VIA is an acceptable, affordable and safe alternative method in developing countries even when compared to the HPV test and cytologic or Pap smears [9, 10]. Visual inspection with acetic acid (VIA) is visualization of woman’s cervix to detect precursors of cervical cancer after application of acetic acid (ordinary table vinegar) on her cervix. Healthcare workers (midwives, nurses and clinical officers) can practice VIA at several levels of healthcare settings [9, 10]. This method also provides an opportunity for simultaneous screening and treatment using cryotherapy at the same visit in low-resource countries in a practice known as “screen and treat” (single-visit approach) [9]. If a VIA test-positive woman is not eligible for cryotherapy because of the extent or placement of a precancerous lesion, loop electrosurgical excision procedure (LEEP) is an alternative treatment option to hysterectomy [11]. Screening with VIA in women at age 35 years or at age 40 years has been shown to reduce lifetime risk of invasive cervical cancer by 25% and by 65% respectively [12].

As in other resource limited countries, VIA is the preferred approach in the Ethiopian population-based cervical cancer screening strategy to detect precancerous cervical lesions [4]. In 2009, the first VIA screening in a single-visit approach was initiated in Ethiopia by the Federal Ministry of Health (FMoH) in collaboration with Pathfinder International. This service was integrated into a comprehensive care package for HIV positive women under the project name of “Addis Tesfa “at 14 public hospitals in the regions of Addis Ababa, Amhara, Oromia, and Tigray, and in the Southern Nations, Nationalities, and People’s Region [12, 13].

Based on the success and lessons learned from this pilot program, Ethiopia’s FMoH plans to scale up the service throughout the country into over 800 health facilities (one health facility per district). The Five-year National Cancer Control Plan of Ethiopia 2016–2020 set a target to achieve at least 80% screening coverage using VIA among all women aged 30–49 years [4]. This target population between 30 years and 49 years of age is the most suitable age to screen women, because the majority of high grade precancerous lesions are detected between these ages. The national protocol should not include women before the age of 30 years in target populations as cervical cancer may take 10–15 years from precancer to invasive cancer as well as the majority of precancerous lesions of cervical cancer at this age will never develop into cancer. Hence, screening younger women will lead to considerable overtreatment, and is not cost-effective. This visual methods also should not include postmenopausal women, because the transformation zone in these women is often inside the endocervical canal and not visible on speculum inspection [4, 9, 10]. To achieve this national target by 2020, VIA screening services have been provided free of charge for the target population since 2016 [4].

The rate of VIA screening positivity (precancerous cervical lesions) among screened women in Ethiopia ranges from 10.3 to 27.7% [14–17], yet evidence on factors associated with utilization of VIA screening service at the community level is still scarce. Thus, the aim of this study was to assess VIA screening service utilization and its associated factors such as sociodemographic characteristics, reproductive and behavioral variables, and awareness of cervical cancer and VIA screening among women aged 30–49 years in Hawassa city, southern Ethiopia. It is hoped that the study findings will provide information to develop and implement strategies to increase the utilization of VIA services for prevention of pre-cervical cancer.

## Methods and materials

### Study design, period and setting

The study was a community based cross-sectional study that was conducted among women aged 30–49 years in Hawassa city from November 15–December 30, 2019. Hawassa is the capital of southern Ethiopia and is located 275 kms south of Addis Ababa (the capital city of Ethiopia). Based on a 2014 projection using the 2007 census, the total number of women aged 30 to 49 years constitutes about 8.9% of a total population of 335,508 [10, 18]. Administratively, Hawassa city is divided into 8 sub-cities with 32 kebeles (smallest administrative units). Hawassa has a total of 28 public health facilities (one referral university hospital, one general hospital, nine health centers and 17 health posts). Four provide VIA screening together with family planning and maternal, newborn and child, and reproductive health services.

### Source and study population

The primary source population was all women aged 30 and 49 years residing in Hawassa city during the study period. The study population included randomly selected women aged 30 and 49 years who were residing in selected kebeles in Hawassa City for at least 6 months and who consented to participate. Women who had had abdominal or vaginal hysterectomy with removal of the uterine cervix were excluded.

### Sample size determination and sampling procedure

A required sample size of 419 was determined using a formula for the single population proportion; n = [(Z _α/2_) ^2^ * (P (1 - P)]/ d^2^ by considering the following assumptions: a 95% confidence level (Zα/2 = 1.96), absolute level of precision or margin of error of 5% (d = 0.5), a 20.9% of proportion of cervical cancer screening (*P* = 0.209) [19], a design effect of 1.5 and a 10% of non-response rate.

A total of 419 study participants were selected using a multistage random sampling technique. Firstly, four sub-cities were randomly selected from the 8 sub-cities. Secondly, one kebele was randomly selected from each selected sub-city. Proportional allocation of sample size for each selected kebele was done based on the number households in each selected kebele. A list of women was obtained from the health extension workers in order to arrange the sample size allocation and the sampling frame of the households. The first household in each kebele was randomly selected, and the remainders of the households were then selected using systematic random sampling with every 13th household to obtain the required sample size for each kebele from the total of 5560 households in four kebeles. If more than one eligible woman lived in the household, only one woman was randomly selected using a lottery method. Eight women were not eligible for this study as two had had a total abdominal hysterectomy with removal of cervix and six were not residents of selected kebeles.

### Ethics approval and consent to participate

Ethical approval was obtained from Hawassa University College of Medicine and Health Sciences Institutional Review Board. Informed consent was obtained prior to each interview. Before obtaining of informed consent from each of the study participants, the purpose of the study was explained for all participants in the study.

### Study variables

The outcome for this study was VIA screening service utilization. Participants were asked whether they ever had VIA screening to detect precursors of cervical cancer after application of acetic acid (ordinary table vinegar) on their cervix and visualized/examined with response options of yes or no. If the possible answer was yes, participants were then asked specific question regarding reasons for undergoing VIA screening. If the possible answer was no, respondents were then asked specific question regarding reasons for not undergoing VIA screening.

The independent variables included sociodemographic variables (marital status (ever/never married), religion (muslim/christian), education (no formal education / primary school/secondary school/college or university), occupation (housewife/self-employee/private employee/civil servant/farmer), ethnicity (sidama/wolayta/amhara/guraghe/other), average monthly household income (1076.9–1722.9 Eth birr (36.7–58.7 USD)/ > 1722.9 Eth birr (> 58.7 USD) / < 1076.9 Eth birr (< 36.7 USD) and age of respondent), reproductive and behavioral characteristics (age at first intercourse (16 and below/above 16), lifetime sexual partners (single/multiple), parity (1–5 /> 5), self-reported HIV sero status (positive/negative), use of oral contraceptive pills (yes /no), history of smoking (yes/no), history of STI (yes/no), family history of cervical cancer (yes/no), known someone with cervical cancer (yes/no), self-perceived risk of developing cervical cancer (yes/no), recived community health education about cervical cancer prevention (yes/no) and received information from health care provider about cervical cancer screening service (yes/no).

Awareness of cervical cancer and VIA screening: Participants who responded yes to the following two questions i.e. “Have you ever heard of cervical cancer?”, “Have you ever heard of VIA screening for cervical cancer?” [20]. Self-perceived risk refers to participants who responded yes to the question “Do you consider yourself at risk for developing cervical cancer” [21]. Received information from health care providers refers to participants who had received advice about cervical cancer screening services from health care provider at health facility level. Community health education refers participants who had received health education from trained community health educators (health extension workers) at home or at a community level about cervical cancer prevention.

### Data collection and data quality control

A pre-tested structured questionnaire was adapted through a review of related literature [22–24], with slight amendments in line with the study objectives and the local context. The questionnaire was initially prepared in English then translated into Amharic and then translated back into English to check for consistency.

Before the actual data collection, the questionnaire was piloted on 21 women in a kebele that was different from the kebeles that had been selected for the study. The questionnaire was then amended to correct any ambiguities or sequencing issues that were discovered during the pilot testing. Four interviewers with diplomas in nursing, and two supervisors with bachelor degree in nursing were trained for 1day on all aspects of the data collection, including-the study objectives and its potential benefits, the questionnaire, interviewing procedures, and maintaining the privacy and confidentiality of the participants. Trained supervisors with experience about VIA services and the principal investigator conducted regular supervision during data collection. Before commencing data analysis, the questionnaire was checked by the principal investigator for data completeness, clarity, and consistency.

### Data processing and analysis

The collected data were coded, entered and cleaned using Epi data version 3.1 and exported to SPSS (Statistical Package for Social Science) version 22 for statistical analysis. Descriptive analysis was conducted. Categorical variables were described by frequencies and percentages while continuous variables were described by means and standard deviations. Bivariate logistic regression and multivariate logistic regression analysis were used to identify associations between VIA screening service utilization and explanatory variables (age of respondents, parity, having history of STI, awareness of cervical cancer, self-perceived risk of developing cervical cancer, receiving advice from health care providers and community health education). Variables associated with VIA screening service utilization in the bivariate logistic regression analysis with a *P* value < 0.2 were included in multivariable logistic regression models.

## Results

### Demographic characteristics of the participants

Four hundred and eleven women were included in the study with a response rate of 98.1%. The mean (± SD) age of the participants was 34.1 ± 6.9 years. A total of 156 (38.0%) women were between the ages 30 to39 years and 255 (62.0%) were aged 40–49. Almost all women, 95.6% were married. About 92.2% of the participants were Christians with the rest being Muslim. One hundred and sixty-four women (39.9%) had no formal education and 26.5% of them had completed only some primary education. About 34.5% of women were housewives and 31.6% were self-employee. Sidama (29.0%), Wolayta (26.0%), Amhara (20.9%) and Guraghe (18.7%) were the major ethnic groups. One hundred and eighty-six 45.26%), 116(28.22%) and 109(26.52%) had earned an average monthly income 1076.90–1722.90 Eth birr (36.7–58.70 USD), > 1722.90 Eth birr (> 58.70 USD) and < 1076.90 Eth birr (< 36.70 USD), respectively (Table 1).

**Table 1:** Socio-demographic characteristics of study participants in Hawassa city, Ethiopia (*n* = 411)

Socio demographic variables	Frequency	Percent
Age (in years)		
30–39	255	62.0
40–49	156	38.0
Marital status		
Ever married	393	95.6
Never married	18	4.4
Religion		
Christian	379	92.2
Muslim	32	7.8
Educational background		
No formal education	164	39.9
Primary school (1–8)	109	26.5
Secondary school (9–12)	80	19.5
College or university	58	14.1
Occupation		
Housewife	142	34.5
Self-employee	130	31.6
Private employee	98	23.8
Civil servant	31	7.6
Farmer	10	2.5
Ethnicity		
Sidama	119	29.0
Wolayta	107	26.0
Amhara	86	20.9
Guraghe	77	18.7
Other^a^	22	5.4
Average monthly household income^b^		
< 1076.9 Eth birr (< 36.7 USD)	109	26.52
1076.9–1722.9 Eth birr (36.7–58.7 USD)	186	45.26
1722.9 Eth birr (> 58.7 USD)	116	28.22

^a^Other ethnicities included Silte, Shekicho, Yem, Kefa, Dawro, Hadiya and Kembata

^b^1 USD was 29.35 Ethiopian birr

Income under extreme poverty < 1.25USD per day

Under poverty1.25-2USD per day

Above poverty line >2USD per day

*ETB* Ethiopian birr, *USD* U. S. dollars

### Reproductive and behavioral related factors

Of the participants, 105 (25.5%) had had sexual intercourse for the first time at age 16 or younger. Approximately 74.2% of study participants had had multiple sexual partners. About 94.9% of women had self-reported being HIV negative and 64.7% had no history of an STI. Two hundred and sixty-two (63.7%) of women had not used oral contraceptives. Only 53 women (12.9%) reported a family history of cervical cancer. Of the participants, 49.9% felt that they were at risk of developing cervical cancer. About 58.2% of the women had received advice on cervical cancer screening from health care provider at health facility level. Two hundred and thirty-nine (58.2%) of women had received health education from trained community health educators (health extension workers) at home or at a community level on cancer prevention (Table 2).

**Table 2:** Reproductive and behavioral characteristics of study participants in Hawassa city, Ethiopia (*n* = 411)

Variables	Frequency	Percent
Age at first sexual intercourse (years)		
16 and below	105	25.5
Above 16	306	74.5
Lifetime number of sexual partners		
Single	106	25.8
Multiple	305	74.2
Parity		
1–5	235	57.2
> 5	176	42.8
Self-reported HIV sero status		
Positive	18	5.1
Negative	338	94.9
Use of oral contraceptive pills		
Yes	149	36.3
No	262	63.7
Ever history of smoking		
Yes	10	2.4
No	401	97.6
Ever history of STI/STD		
Yes	145	35.3
No	266	64.7
Family history of cervical cancer		
No	53	12.9
Yes	358	87.1
Knew someone with cervical cancer		
Yes	43	10.5
No	368	89.5
Self-perceived risk of developing cervical cancer		
Yes	205	49.9
No	206	50.1
Getting advice from health care providers		
Yes	239	58.2
No	172	41.8
Getting community health education		
Yes	239	58.2
No	172	41.8

### Awareness of cervical cancer and VIA service utilization

Although 72.5% of the surveyed participants who had heard about cervical cancer, only 37.5% of whom had heard about VIA screening. Overall, 37.5% of the respondents were aware of cervical cancer and VIA screening. Eighty-five (20.7%) of the participants reported having had cervical cancer screening though VIA. The common reasons for undergoing VIA screening were at the suggestion of health care workers (49.4%), a result of a screening campaign (33.0%) and for medical reasons (abnormal vaginal bleeding and pelvic pain) 17.6%. The most common reason for not undergoing VIA screening was lack of awareness (34.4%), followed by absence of medical reasons (27.0%). Other reasons were fear of test results (19.0%), the cost of screening (12.3%) and lack of knowledge about where VIA screenings took place (7.4%) (Table 3).

**Table 3:** Awareness status, screening utilization and participants’ suggestion for improving screening service among study respondents in Hawassa city, Ethiopia (*n* = 411)

Variables	Frequency	Percent
Ever heard of cervical cancer		
Yes	298	72.5
No	113	27.5
Ever heard of VIA screening		
Yes	154	37.5
No	257	62.5
Ever had VIA screening		
Yes	85	20.7
No	326	79.3
Reasons given for undergoing VIA screening (*n* = 85)		
Health care provider suggestion	42	49.4
Screening campaign	28	33.0
Medical reasons (abnormal vaginal bleeding or pain)	15	17.6
Reasons given for never being screened (*n* = 326)		
Lack of awareness	112	34.4
Absence of any symptoms/abnormality	88	27.0
Fear of test outcome	62	19.0
Cost of service	40	12.3
Lack of knowledge the place of VIA screening	24	7.4
Participants suggestion for improving the VIA service		
Increasing health education	229	55.7
Community screening campaign	70	17.0
Improving service provision	125	30.4

### Factors associated with VIA service utilization

In bivariate logistic regression analysis, age of respondents (OR = 1.73, 95% CI: 1.03–2.91), parity (OR = 0.41, 95% CI: 0.25–0.70), having history of STI (OR = 4.50, 95% CI: 2.72–7.44), awareness of cervical cancer and VIA screening (OR = 1.19, 95% CI: 0.73–1.93), self-perceived risk of developing cervical cancer (OR = 2.80, 95% CI: 1.68–4.66), received advice from health care providers (OR = 3.63, 95% CI: 2.04–6.44) and community health education (OR = 1.61, 95% CI: 0.98–2.66) were factors associated with VIA screening service utilization. However, education, occupation, monthly income, age at first intercourse, lifetime sexual partners, self-reported HIV sero status, use of oral contraceptive, history of smoking, family history of cervical cancer and knew someone with cervical cancer has were not associated with VIA screening service utilization. In the multivariable logistic analysis, age of the participants, history of STI, awareness of cervical cancer and VIA screening, self-perceived risk of developing cervical cancer, having received information from health care providers and community health education remained significantly associated with VIA screening service utilization.

The odds ratio of use of VIA screening were almost 5 times higher (AOR = 4.64, 95%CI: 2.15–10.01) in those study participants aged 40-49 years when compared to those aged 30–39 years. The odds of VIA screening were about 4 times higher (AOR = 3.90, 95%CI: 2.02–7.53) among those study participants with a history of STI when compared to those without a history. The odds of use of VIA screening were about 4.5 times higher (AOR = 4.52, 95%CI: 1.69–12.11) among those participants who had received information from health care providers when compared to those who had not received information from health care providers. Moreover, the odds of use of VIA screening were about 4 times higher (AOR = 3.67, 95%CI: 1.68–8.04) among those participants who had awareness about cervical cancer and VIA screening when compared to those who had no awareness about cervical cancer or VIA screening. The use of VIA screening was about 3.5 times higher (AOR = 3.52, 95%CI: 1.74–7.13) among those with a self-perceived risk of developing cervical cancer when compared to those without a self-perceived risk. In this study, the odds of use of VIA screening were about 6 times higher (AOR = 6.25, 95%CI: 2.99–13.05) among those study participants with community health education when compared to those without community health education (Table 4).

**Table 4:** Bivariable and multivariable analysis of factors associated with VIA service utilization among study participants in Hawassa city, Ethiopia (*n* = 411)

Variables	Screened with VIA N (%)	Unadjusted odds ratio (95% CI)	Adjusted odds ratio (95%CI)	*p* value
Age				
30–39	24(15.4)	1.00	1.00	.000
40–49	61 (23.9)	1.73 (1. 03–2.91)	4.64 (2. 15–10.01)	
Parity				
1–5	19(10.8)	1.00	1.00	.287
> 5	66(28.1)	3.23 (1. 85–5.62)	0.59 (0. 23–1.55)	
Having history of STIs				
No	31 (11.7)	1.00	1.00	.000
Yes	54 (37.2)	4.50 (2. 72–7.44)	3.90 (2. 02–7.53)	
Advice from health care providers				
No	17 (9.9)	1.00	1.00	.003
Yes	68(28.5)	3.63 (2. 04–6.44)	4.52 (1. 70–12.11)	
Awareness on VIA screening				
No	50 (19.6)	1.00	1.00	.001
Yes	35 (22.4)	1.19 (0. 73–1.93)	3.67 (1. 68–8.04)	
Self-perceived risk of developing cervical cancer				
No	26(12.6)	1.00	1.00	.000
Yes	59(28.8)	2.80 (1. 70–4.66)	3.52 (1. 74–7.13)	
Community health education				
No	28 (16.3)	1.00	1.00	.000
Yes	57 (23.8)	0.06 (1. 61–0.10)	6.25 (2. 10–13.05)	

### Suggestion for improving utilization of VIA

In the present study, participants suggested increasing community health education (55.7%), community screening campaigns (17.0%) and improving VIA screening service provision (30.4%) to improve VIA screening service utilization in their communities (Table 3).

## Discussion

The extent of self-reported use of VIA screening among women aged 30–49 was just 20.7% in current study. This level of screening is much lower than the national cancer control plan of Ethiopia’s target level of 80% [4], and lower than found in studies conducted in Jamaica (66%) [25] and Kenya (39%) [26]. However, it was greater than found in other studies conducted in Ethiopia (2.9%) [27] and Uganda (4.8%) [28]. The reason for this disparity might include differences in screening methods, the study populations, and the varying socio-demographic characteristics of study participants, as well as in the accessibility of screening services and availability of trained health workers. Access to mass media in an urban population could also be a factor.

This study showed that those women who were older aged 40–49 years, were 4.64 times more likely to be screened through VIA when compared to participants aged between 30 and 39 years. This finding was supported by previous studies conducted in Ethiopia [27, 29]. This high likelihood of screening might be because as a woman’s age increases, she might have experienced increased exposure to reproductive health care, antenatal care, delivery and post-natal care from health facilities. As a result, she might have had more opportunities to receive health education and counseling from health workers on cervical cancer and the screening service.

Women who reported a history of sexually transmitted disease were more likely to be screened than those participants who had no history of sexually transmitted disease. This finding was supported by studies conducted in Mekele, Ethiopia [29] and Kenya [30]. Women who have had STIs may have been given information about the connection between cervical cancer and STIs from health care providers during the time of their STI treatment at a health facility.

Those women who had been informed about cervical cancer screening by a health care provider were more likely to have utilized VIA screening when compared with women who had never been informed about screening. This finding was supported by a study conducted in rural Uganda [31], and suggests that information from health care providers creates an awareness of cervical cancer and the purpose of VIA screening services, which in turn increases the likelihood of seeking screening services. Awareness of cervical cancer and screening services was also associated with cervical cancer screening utilization as was found in a study conducted in Nigeria [31]. Women who had awareness about cervical cancer and screening service might have increased self-perceived risk of developing cervical cancer and this knowledge may have encouraged women to seek screening service, increasing screening service utilization.

Women who felt more perceived risk of developing cervical cancer were more likely to undergo screening as compared to those participants who had no self-perceived risk of developing cervical cancer. This finding was supported by studies conducted in Kenya [32] and Thailand [33]. Women who had self- perceived susceptibility for developing cervical cancer might be more willing to use screening service, in order to protect themselves from cervical cancer.

Those women who had received health education about cervical cancer prevention from trained community health educators (health extension workers) were more likely to undergo screening when compared to those who had not received health education. This finding was supported by Nigerian studies [34, 35]. Women who have participated in community health education might have a better awareness and knowledge about cervical cancer risk and the value of screening, which in turn may improve the uptake of cervical cancer screening.

A strength of this study is that we used a community-based study in order to increase generalizability. Moreover, this study used systematic random selection of participants in the community in order to generate accurate estimates representative of the population. Limitations of this study include that it was unable to measure the causal relationship between exposure and outcome due its cross-sectional study design. In addition, this study was conducted in an urban community in Hawassa city, so the findings are not generalizable to rural areas or other parts of Ethiopia. Finally, social desirability bias may have affected the results as the information was self-reported.

## Conclusion

The extent of self-reported use of VIA screening was low when compared to National Cancer Control Plan of Ethiopia 2016–2020 target levels. Several factors including older age, history of STI, awareness of cervical cancer and VIA screening, self-perceived risk of developing cervical cancer, receiving advice from healthcare workers and community health education were significantly associated with VIA screening service utilization. The study found that there is a need for increased health education-campaigns at both the community level and in healthcare facilities in order to create awareness about the importance of cervical cancer screening services and to decrease the risk of cervical cancer. Further research should be conducted to investigate awareness about the risks of cervical cancer and use of VIA screening service among the higher-risk groups of younger women who had immune suppression (HIV) and early initiation of sexual activity.

## Data Availability

All the required data are available in the main document.

## Abbreviations

AOR: Adjusted Odd Ratio
OR: Odd Ratio
HIV: Human Immunodeficiency Virus
HPV: Human Papilloma Virus
SD: Standard deviation
SPSS: Statistical Package for Social Sciences
STI: Sexually Transmitted Infection
VIA: Visual Inspection with Acetic acid
